# Research on Two-Layer Polymer Composites Alternatively Obtained in a Constant Magnetic Field

**DOI:** 10.3390/ma18020255

**Published:** 2025-01-09

**Authors:** Ewa Miękoś, Marek Zieliński, Michał Cichomski, Tomasz Klepka, Dorota Czarnecka-Komorowska, Dominika Drzewiecka, Dariusz Sroczyński, Anna Fenyk

**Affiliations:** 1 Department of Inorganic and Analytical Chemistry, Faculty of Chemistry, University of Lodz, Tamka 12, 91-403 Lodz, Poland; marek.zielinski@chemia.uni.lodz.pl (M.Z.); dariusz.sroczynski@chemia.uni.lodz.pl (D.S.); anna.fenyk@chemia.uni.lodz.pl (A.F.); 2 Department of Materials Technology and Chemistry, Faculty of Chemistry, University of Lodz, Pomorska 163, 90-236 Lodz, Poland; michal.cichomski@chemia.uni.lodz.pl; 3 Department of Technology and Polymer Processing, Faculty of Mechanical Engineering, Lublin University of Technology, Nadbystrzycka 36, 20-618 Lublin, Poland; t.klepka@pollub.pl; 4 Department of Plastics Division, Institute of Materials Technology, Poznan University of Technology, Piotrowo 3, 61-136 Poznan, Poland; dorota.czarnecka-komorowska@put.poznan.pl; 5 Department of Biology of Bacteria, Faculty of Biology and Environmental Protection, University of Lodz, Banacha 12/16, 90-237 Lodz, Poland; dominika.drzewiecka@biol.uni.lodz.pl

**Keywords:** polymers, composites, constant magnetic field (CMF), *Curcuma longa* L., *Solidago virgaurea* L.

## Abstract

The aim of this research was to obtain two-layer polymer composites with favorable mechanical and functional properties. The composites consisted of one lower layer of polymer with less elastic properties, containing no admixtures, and one upper layer of polymer with more elastic properties, containing plant admixtures, in the amount of 10% by weight of either goldenrod (*Solidago virgaurea* L.), or of turmeric (*Curcuma longa* L.). The admixtures *S*. *virgaurea* and *C. longa* were intended to introduce new biodegradable and medicinal properties without causing too much deterioration of physical or mechanical properties. Some polymer composites additionally contained magnetic particles in the form of carbonyl iron (Fe) in the amount of 20% by weight. The tests of mechanical tensile strength of the composites, water absorption, frost resistance, and surface contact angle were performed. Microscopic examinations determined the roughness of the cross-sectional surfaces. A constant magnetic field with magnetic induction B, which was an additional external factor changing the properties and structure of two-layer polymer composites, was also used in the research.

## 1. Introduction

Two-layer polymer composites are used in the chemical, construction, and electrical industries, among others, and new materials with properties meeting the current requirements are being sought.

Miękoś E. et al. [1] investigated polymer composites based on polylactide (PLA) and epoxy resin in terms of the effect of a constant magnetic field on changes in their physical and chemical properties. The composites contained admixtures in the form of magnetite (Fe_3_O_4_) and crystalline cellulose in the amounts of 10%, 20%, and 30% by weight, as well as starch in the amount of 10%. The introduction of fillers in the form of cellulose and starch was aimed at obtaining partially biodegradable composites. Changes in the properties of composites obtained in a constant magnetic field with a magnetic induction value of B = 0.5 T were observed. SEM microscopy studies were also carried out; the composition of the composites was determined by EDS, and their structure was examined using the XRD method. On the basis of the obtained results, it was found that the properties of the composites change depending on the value of magnetic induction.

Also, Miękoś E. et al. [2] and Fu Y. [3] investigated the effect of a constant magnetic field on polymer composites whose matrix was epoxy resin with other fillers, mainly of natural origin. The filler used was birch bark, which contains betulin, i.e., a compound with bactericidal and virucidal properties, and yellow dextrin in mass quantities of 20%. Expanded graphite and powder graphite (20% *w*/*w*) were also used as admixtures. The recorded increase in the hardness of the composite with the addition of birch bark was approx. 12%—from 24.01 to 26.96 N/mm^2^, and in the case of the composite with the addition of yellow dextrin approx. 15% by weight, from 26.12 to 29.93 N/mm^2^.

Zhao et al. [4] worked on improving the materials used to manufacture medical devices, using a new composite material. The aim of the study was to prepare a versatile composite by designing polyurethanes with an ion chain extender and mixing them with cellulose. The composite demonstrated exceptional properties, such as excellent mechanical strength and high bactericidal activity. In their review article, De Oliveira Filho et al. [5] reported curcumin to be a multifunctional molecule, which can be successfully used in the production of all types of food packaging. The material developed with the use of curcumin could be a smart packaging solution for monitoring the quality of food products such as meat, seafood, and beverages. Active packaging can exhibit antioxidant and antimicrobial effects, which can improve the quality and preservation of food products.

A composite film based on chitosan and polyvinyl alcohol mixed with turmeric powder for use in food packaging was developed by Bhat et al. [6]. Active chitosan and polyvinyl alcohol films with varying concentrations of turmeric powder were prepared by solvent casting. The results demonstrated an improvement in the mechanical and antibacterial properties of all the films produced. All of them were biodegradable under environmental conditions. The results of the study suggest that the developed films can be used in the food packaging industry to increase the safety and quality of packaging materials.

A similar role can be played by inorganic nanoparticles, in particular metal oxide nanoparticles, which have attracted considerable attention precisely because of their strong bactericidal effect [7].

Kheira et al. [8] used metal oxide nanoparticles to produce nanocomposite materials. They described a simple method for manufacturing antibacterial nanocomposites doped with Ag, TiO_2_, and ZnO, which were dispersed in a liquid silicone rubber structure at four concentrations. The nanocomposites prepared on the basis of silicone rubber—15% *w*/*w* TiO_2_—demonstrated the best antibacterial efficacy. In addition, the TiO_2_-doped nanocomposites formed the stiffest nanocomposites with a very fine, even surface, which increased the surface hydrophobicity, preventing further bacterial growth.

In the available state of the art, there is no way to correct the results of tensile strength tests for two-layer polymer composites in each polymer layer on the basis of tensile strength measurements and stress measurements of both polymer layers of the composite at the same time, without the need to separate them. In paper [9], the authors presented a developed mathematical model for determining the sequence of layer damages in a two-layer ceramic composite as well as for determining the conditions for achieving the maximum limit load with three-point loading. The adequacy of the obtained model was confirmed by the results of experimental tests involving the three-point bending of experimental samples. The obtained results allow us to predict in advance, based on the parameters of both layers, which layer will trigger the cracking mechanism.

The methodology for determining the strength values of composite samples was revealed in paper [10]. It was a review of the literature on the management methods of outlier results, analyzing the possible causes of the occurrence of outlier test results in composite materials. The research was carried out on the results of two series of samples: one from the tensile test and one from the shear test, additionally differing in the sample size. In that paper, the dispersion of values analyzed during compression and shear tests was verified. To indicate the outliers, the maximum normalized MNR (Maximum Normed Residual) method was used. For each of the samples, the MNR was calculated and compared with the cV (critical value), corresponding to the sample size of the particular sample series.

Paper [11] presents a simplified approach to the correlation of experimental tensile strength values of randomly oriented composites based on polymers reinforced with short fibers (SFs). For this purpose, a model correlating the experimental tensile strength values of SF-reinforced composites was derived. The model was derived on the basis of the mixture rule. The experimental strength of the composite was found to be significantly dependent on the interfacial stress on the contact surface of the matrix and the percentage of fibers oriented in the direction of tension.

The aim of the research described in paper [12] was to model the behavior of the two-layer system of the composite material during its production. Using the developed model, a study of changes in the stress and strain state was carried out. Numerical modeling was carried out for two types of two-layer materials combined in a similar way. When two-layer bonded materials are produced, there is always a primary deformation that occurs on the bottom plate of the lower layer. However, the maximum plastic deformation will be represented for a layer with a lower value of the modulus of elasticity.

Multilayer composite structures are characterized by high damping efficiency, which has wide application prospects in the aerospace and mechanical engineering industries.

In paper [13], two formulas are derived to predict the tensile strength of composites. These two formulas reflect that the tensile strength of composites is a function of the volume fraction of the fibers.

Paper [14] proposes the Monte Carlo method in combination with a random probability model for the calculation of the longitudinal tensile strength of unidirectional composites. This method takes into account the two-dimensional distribution of fibers in the cross-section, while the theoretical analysis method takes into account only the linear distribution of fibers. The results show good consistency of the calculated longitudinal tensile strength values of the composites with the experimental results.

This paper presents the obtained mechanical parameters, water absorption, frost resistance, and surface roughness of the cross-sections of two-layer polymer composites. It has also been indicated what effect a constant magnetic field has on these parameters. A method of correction of the results of tensile strength test, according to the DIN EN ISO 527-1 standard [15], has also been developed for two-layer polymer composites. On the basis of the research, a relationship was developed to correct the stress sought—σ—that arises during tension in a two-layer composite simultaneously in both polymer layers of the composite, without the need to separate them.

## 2. Results and Discussion

### 2.1. Testing of Mechanical Properties

#### 2.1.1. The Development of a Relationship Correcting the Stress σ That Arises During Tension Simultaneously in Both Polymer Layers of a Two-Layer Composite, Without the Need to Separate Them

**Figure 1:**
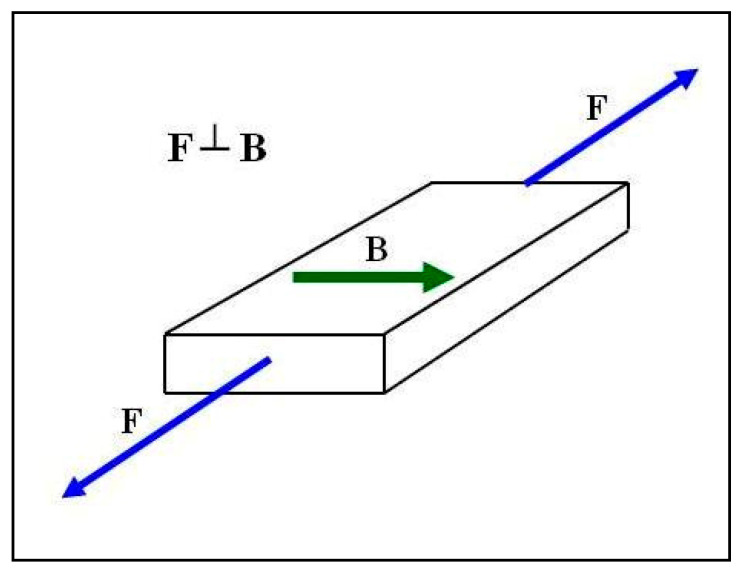
Mechanical stretching method of polymer composites.

Paper [4] shows that composites achieve anisotropic properties in a constant magnetic field. The anisotropic properties depend on the direction used when testing these features; hence, the method of stretching the polymer composites was selected in accordance with the one that provided more favorable values, i.e., the tensile forces F were directed perpendicular to the direction of action of the magnetic induction vector B during the polymerization of the composite (Figure 1).

**Figure 2:**
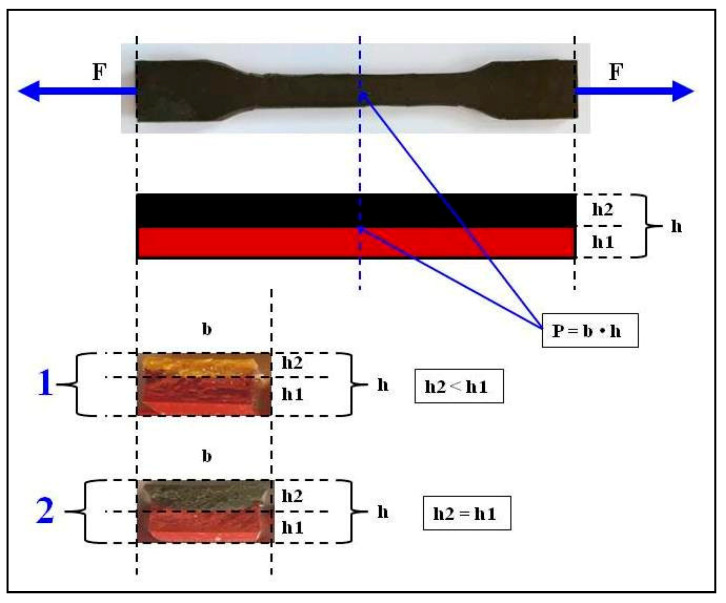
Molds of two-layer polymer composites designed for tensile strength tests in accordance with the DIN EN ISO 527-1 standard, where F—tensile forces; h1—the height of the lower layer; h2—the height of the upper layer; P—cross-sectional area; and b—the thickness of the constriction. Composite 1 contains different thicknesses of polymer layers (h2 < h1) and composite 2 contains two equally thick polymer layers (h2 = h1).

The magnetic particles of carbonyl iron (Fe) under a constant magnetic field were arranged in chains in the direction of the magnetic induction vector B, creating additional build-up and reinforcement of the composite. The disruption of such a sample required more force. Polymer composite molds were prepared in accordance with the DIN EN ISO 527-1 standard (Figure 2).

The first bottom layer was made of pure silicone rubber “Polastosil M-56”, which is less elastic. After the polymerization period, the second, upper layer of silicone rubber “Gumosil B” molds, with greater elasticity and turmeric powder—*C. longa*, was prepared. The molds had the following dimensions: the total height of the composite sample was h = 4.22 mm, which was the sum of individual layers h1 and h2, and the width of the narrowed portion of composite sample was b = 10 mm. The surface area P that was breaking was therefore the product of b and h. Thus, the stresses σ that formed in the composite material during tension were the quotient of the tensile force F and the surface area P that was being broken.

The tensile stress read from the device for composite with a turmeric powder content of 10% *w*/*w* was σcur = 1.153359 MPa. Using the correction Equation (8) given in Section 3.3, Testing Methodology, and the calculated constant, it was possible to obtain the corrected stress value of this composite:

(1) σ cur=Fcur·h2h1(h1+h2)·1.0957683

where

(2) Fcur=σ cur·Pcur=σ curb·h=1.153359 MPa·0.422 cm2=4.9631361 kG

where h1 = 0.2532 cm, and h2 = 0.1688 cm.

Thus, the value of the adjusted stress was:

(3) σ cur=4.9631361·0.16880.2532(0.2532+0.1688)·1.0957683=8.5915429kGcm2=0.8425425 MPa

Equation (3) demonstrates that the corrected value is much lower than one read from the device. Thus, if h1 ≠ h2, the calculation of tensile stress without correction would result in an error of 26.9%.

#### 2.1.2. Studies of the Correlations of Tensile Stresses σ_m_ and Deformations ε_m_ of Polymer Composites with the Applied Magnetic Induction B

Composites with an admixture of turmeric powder—*C. longa*—and carbonyl iron with magnetic properties were used in the research. A constant magnetic field was used in some samples during polymerization. The samples of polymer composites numbered from 4 to 9 were tested (Table 1).

**Figure 3:**
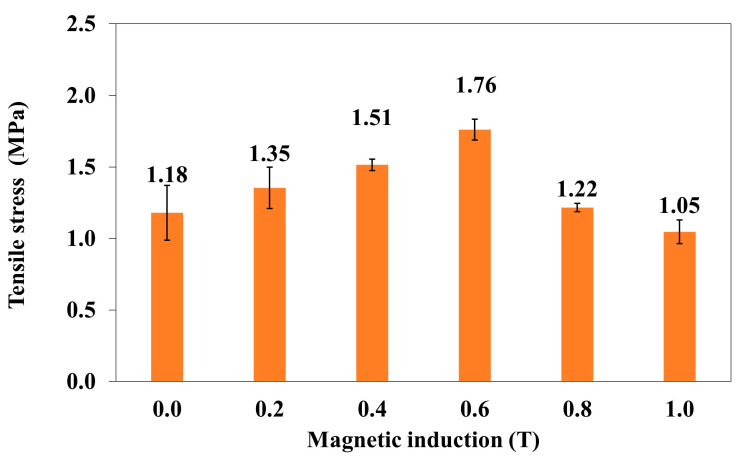
The tensile stress (σ_m_) of the polymer composite (silicone rubber + Fe + turmeric powder) as a function of magnetic induction B.

The dependence of the tensile stress (σ_m_) of the polymer composite on magnetic induction B is shown in the graph (Figure 3). 

An increase in the induction of the magnetic field of B to 0.6 T causes an increase in tensile stress in the polymer composite. A further increase in B causes the destruction of the composite “reinforcement” of magnetic particles, making the composite sample less resistant to tensile stresses. The effect of the magnetic field on the atoms manifests itself in the form of stresses in the crystal lattice. In a liquid, i.e., for instance, before the polymerization of the composite, magnetic fields acting on both electrons and ionized atoms cause dynamic effects, one of which is the volumetric movement of the medium. The movement of the masses, in turn, causes the modification of the fields. Thus, we are dealing with a complex coupled system of matter and fields. With the influence of a magnetic field, hydrodynamic forces become important.

As a result of these interactions, a magnetohydrodynamic force, known as the Lorentz force, is generated in the solution before the polymerization of the composite. Polymer composites with carbonyl iron display anisotropic properties. The method of stretching of polymer composites was chosen to obtain more favorable values, i.e., the tensile force F was perpendicular to the direction of the magnetic induction vector B (Figure 1). The higher value of magnetic induction B and the lower distance between chains of the carbonyl iron particles arranged perpendicularly to vector B lead to a more uniform internal structure. Thus, it is necessary to apply a higher tensile force F in order to break the composite sample, which in turn leads to an increase in the stress δ in the sample. The following prerequisite must be met: the surface area of the sample rupture P is constant according to the formula δ = F/P (Figure 2). The tested composites are elastic materials and, at a critical moment, in the case of composites under investigation at magnetic induction B = 0.6 T, the sample was deformed and the rupture surface P was decreased. Therefore, a lower force F is needed to completely break the sample with the smaller surface area P, which is equivalent to a lower stress δ in the sample. The samples were stretched with the same speed of 50 mm/min, and thus, the time and stress at which a decrease in the rupture surface P was followed by the breaking of the sample were unique for each sample.

In order to determine a statistical effect of magnetic field induction B on the tensile stress of polymer composite (silicone rubber + Fe + turmeric powder), an ANOVA analysis was performed. It was found that the *p*-value was lower than 0.05, which means that there are statistically significant differences between results. Thus, one can conclude that magnetic field induction B has a statistically significant effect on the tensile stress of this composite.

**Figure 4:**
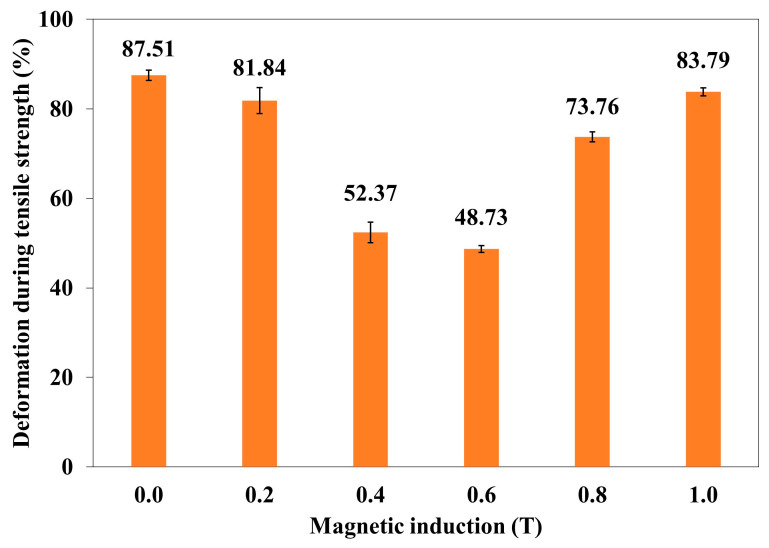
Deformation during tensile strength (ε_m_) of polymer composite (silicone rubber + Fe + turmeric powder) as a function of magnetic induction B.

The correlation of deformation (ε_m_) of the polymer composite with magnetic induction B is shown in the graph (Figure 4).

An increase in the induction of the magnetic field B to 0.6 T results in a decrease in the deformation in the polymer composite. It is the process opposite to increasing tensile stress. A further increase in B causes the deformation of the composite to increase again.

In order to determine a statistical effect of magnetic field induction B on deformation during tensile strength of polymer composite (silicone rubber + Fe + turmeric powder), an ANOVA analysis was performed. It was found that *p*-value was lower than 0.05 which means that there are statistically significant differences between results. Thus, one can conclude that magnetic field induction B has a statistically significant effect on tensile strength during the deformation of this composite.

### 2.2. Water Absorbability and Frost Resistance

**Figure 5:**
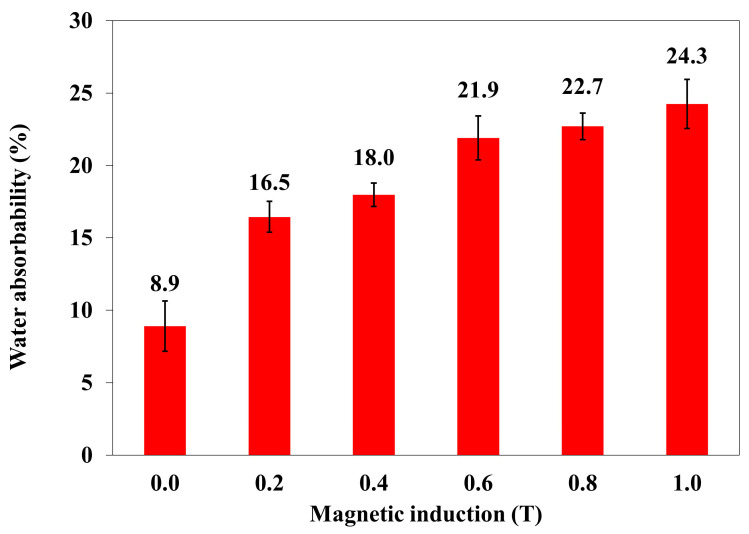
The water absorbability of polymer composites with turmeric powder as an admixture (powder, 10% *w*/*w*) and carbonyl iron (20%) as a function of magnetic induction B [water absorbability (% × 10^−3^)].

Water absorption and frost resistance tests were also carried out on polymer composites with an admixture of turmeric powder and carbonyl iron. These were composite samples numbered from 4 to 9 (Table 1). Figure 5 shows the dependence of water absorption on the applied magnetic induction B.

An increase in the value of magnetic induction B causes an increase in the water absorbability of polymer composites with the addition of turmeric powder (10% *w*/*w*) and carbonyl iron (20%). The arrangement of the composite particles in the direction of the magnetic induction vector results in the appearance of additional spaces (small channels), making it possible for water to penetrate into the composite.

In order to determine a statistical effect of magnetic field induction B on the water absorbability of the polymer composite with turmeric powder as an admixture (powder, 10% *w*/*w*) and carbonyl iron (20%), an ANOVA analysis was performed. It was found that the *p*-value was lower than 0.05, which means that there are statistically significant differences between results. Thus, one can conclude that magnetic field induction B has a statistically significant effect on the water absorbability of this composite.

**Figure 6:**
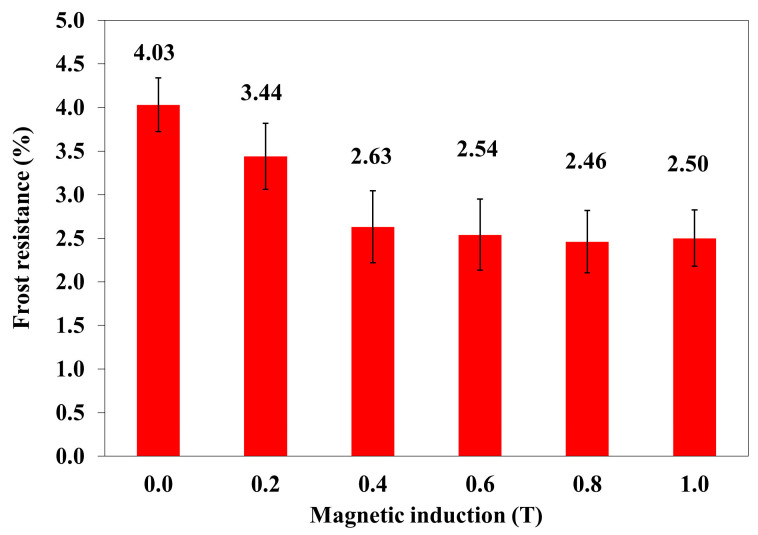
The frost resistance of polymer composites with turmeric powder (10% *w*/*w*) and carbonyl iron (20%) as a function of magnetic induction B.

The frost resistance of composites is the loss of their mass as a result of the cyclic freezing and thawing of the samples in water. Figure 6 shows the dependence of frost resistance on magnetic induction B.

The increase in the value of magnetic induction B resulted in an improvement in the frost resistance of polymer composites, i.e., reduced weight loss and its stabilization at a certain level. If magnetic induction B increases, the energy of the magnetic field Em acting on the polymer composite increases exponentially.

In order to determine a statistical effect of magnetic field induction B on frost resistance of polymer composites with turmeric powder (10% *w*/*w*) and carbonyl iron (20%), an ANOVA analysis was performed. It was found that the *p*-value was lower than 0.05, which means that there are statistically significant differences between results. Thus, one can conclude that magnetic field induction B has a statistically significant effect on the frost resistance of this composite.

### 2.3. Surface Water Contact Angle

**Figure 7:**
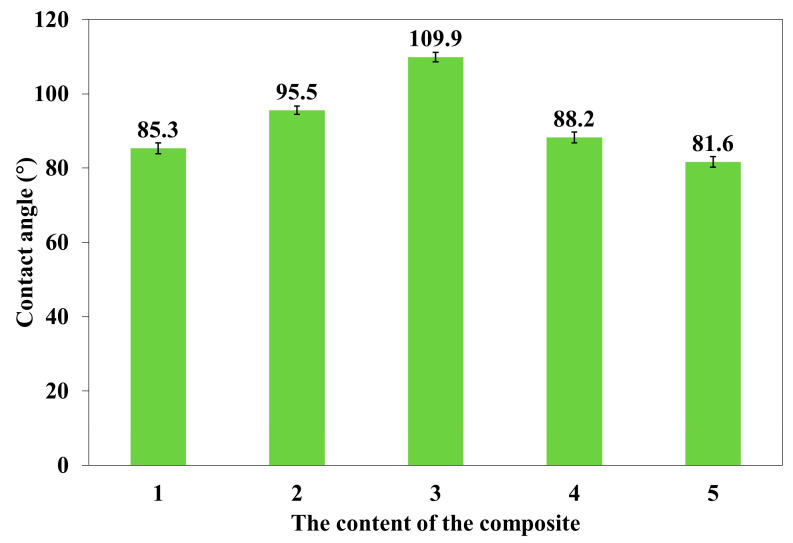
The dependence of the surface contact angle of polymer composites on the composite number, i.e., the applied admixture and the applied magnetic induction B, where 1. Silicone rubber (B = 0); 3. Silicone rubber and *C. longa* (B = 0); 4. Silicone rubber and *C. longa* and carbonyl iron (B = 0); 5. Silicone rubber and *C. longa* and carbonyl iron (B = 0.2 T); and 7. Silicone rubber and *C. longa* and carbonyl iron (B = 0.6 T).

In the studies of the contact angle of the surface, polymer composites numbered 1, 3, 4, 5, and 7 (Table 1) were used. The dependence of the surface contact angle (°) of polymer composites on the composite number, i.e., the applied admixture and the applied magnetic induction B, is shown in Figure 7.

A constant magnetic field of B = 0.2 or 0.6 T reduced the surface contact angle of polymer composites. Polymer composites, in accordance with the principle, became more hydrophilic. The above is consistent with studies in which a constant magnetic field increased the absorbability of polymer composites.

In order to determine the statistical effect of magnetic field induction B on surface contact angle of polymer composites 4, 5, and 7, an ANOVA analysis was performed. It was found that the *p*-value was lower than 0.05, which means that there are statistically significant differences between results. Thus, one can conclude that magnetic field induction B has a statistically significant effect on surface contact angle of these composites.

### 2.4. The Results of Roughness Measurements of the Cross-Sectional Surfaces of the Composites

A cross-section of the composite was obtained by cutting the composite sample with a surgical blade from Swann-Morton Ltd. (Sheffield, UK). Images and measurements of surface roughness of the cross-sections of the composites were made using the UP-3000 optical 3D profilometer from Rtec-Instruments Inc. (San Jose, CA, USA). The images and surface roughness measurements were obtained at 20× magnification with a field of view of 860 μm × 650 μm, an optical resolution of 0.31 μm, and a vertical resolution of 8 nm. The surface roughness parameters defined in the ISO 25178 standard [16] were calculated using MountainsMap Imaging Topography ver. 9.3 software (Digital Surf, Besançon, France). The following parameters were calculated in (μm): Sq (the root mean square height of the surface); Sp (the distance of the highest point of the surface from the mean plane) (maximum height of peaks); Sv (the distance of the lowest point of the surface from the mean plane) (maximum height of valleys); Sz (the maximum height of the surface—sum of Sp + Sv) (the maximum height of the surface); and Sa (the arithmetical mean height of the surface from the mean plane). Six samples numbered 1, 2, 3, 4, 5, and 7 (Table 1) were examined in three parts: with respect to the lack of admixtures, with respect to the type of admixtures, and with respect to the additionally applied constant magnetic field.

**Figure 8:**
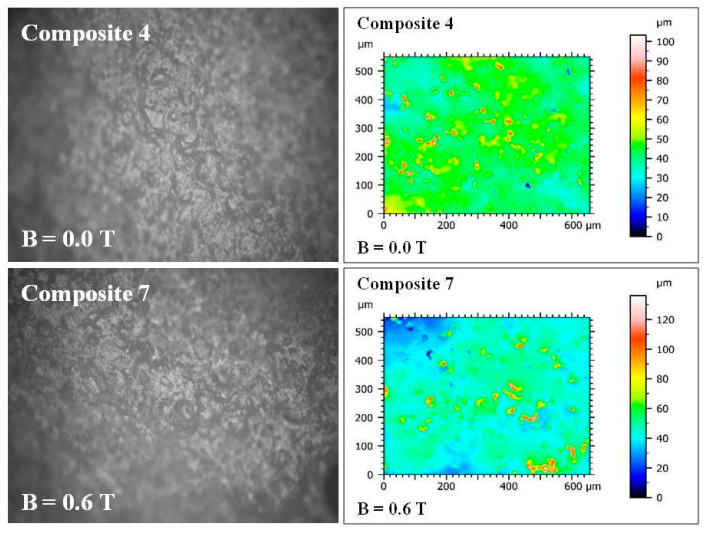
Images of the cross-sectional surfaces of composites No. 4 and 7 taken with the UP-3000 3D optical profilometer from Rtec-Instruments Inc. (San Jose, CA, USA). Taken at 20× magnification with field of view of 860 μm × 650 μm, 0.31 m optical resolution μ, and 8 nm vertical resolution.

The first part concerned the change in parameters in the bottom layer of polymer composites (Polastosil M-56), where there are no admixtures. In a constant magnetic field, changes in parameters ranging from a few to a dozen or so μm were visible. Composites No. 4 (B = 0) and No. 7 (B = 0.6 T) were compared (Figure 8).

As a result of the application of a constant magnetic field of B = 0.6 T during polymerization, all roughness parameters increased: Sq (the root mean square height of the surface) from 6.71 to 10.80 μm; Sp (the maximum height of peaks) from 59.76 to 90.23 μm; Sv (the maximum height of valleys) from 43.47 to 45.85 μm; Sz (the maximum height of the surface) from 103.24 to 136.09 μm; and Sa (the arithmetical mean height of the surface) from 4.45 to 6.43 μm. This clearly indicates an increase in roughness due to the effect of constant magnetic field on the lower layer of the composite (Polastosil M-56), despite the lack of impurities.

**Figure 9:**
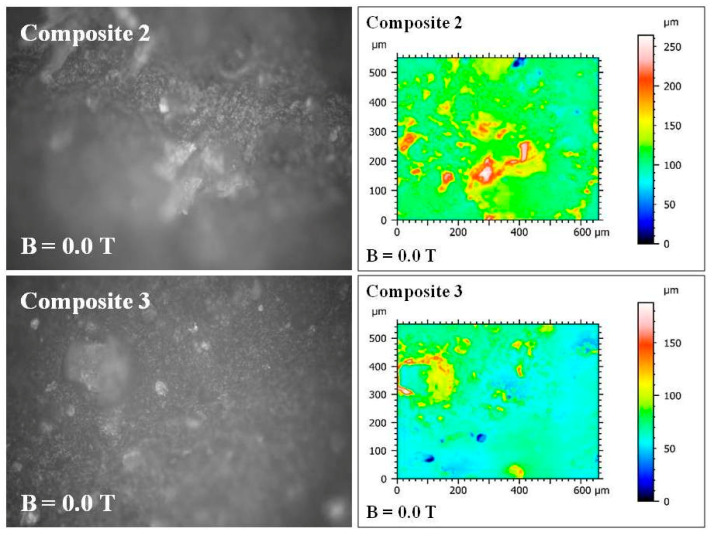
Images of the cross-sectional surfaces of composites No. 2 and 3 taken with the UP-3000 3D optical profilometer from Rtec-Instruments Inc. (San Jose, CA, USA). Taken at 20× magnification with field of view of 860 μm × 650 μm, 0.31 m optical resolution μ, and 8 nm vertical resolution.

The second part concerned the change in parameters in composites in the upper layer (Gumosil B), where admixtures are used. The filler change from *S. virgaurea* to *C. longa* caused large changes in these parameters, despite the lack of a constant magnetic field. Composites No. 2 and No. 3 were compared, both obtained without the participation of a constant magnetic field (Figure 9).

In that case, during polymerization, all roughness parameters were reduced as a result of changing the filler from *S. virgaurea* to *C. longa*: Sq (the root mean square height of the surface) from 24.43 to 15.11 μm; Sp (the maximum height of peaks) from 146.59 to 120.96 μm; Sv (the maximum height of valleys) from 117.73 to 66.98 μm; Sz (the maximum height of the surface) from 264.32 to 187.94 μm; Sa (the arithmetical mean height of the surface) from 16.71 to 10.21 μm. In the upper layer of the composite (Gumosil B), where the admixtures were located, the roughness of the polymer composite decreased as a result of such a change in the admixture.

**Figure 10:**
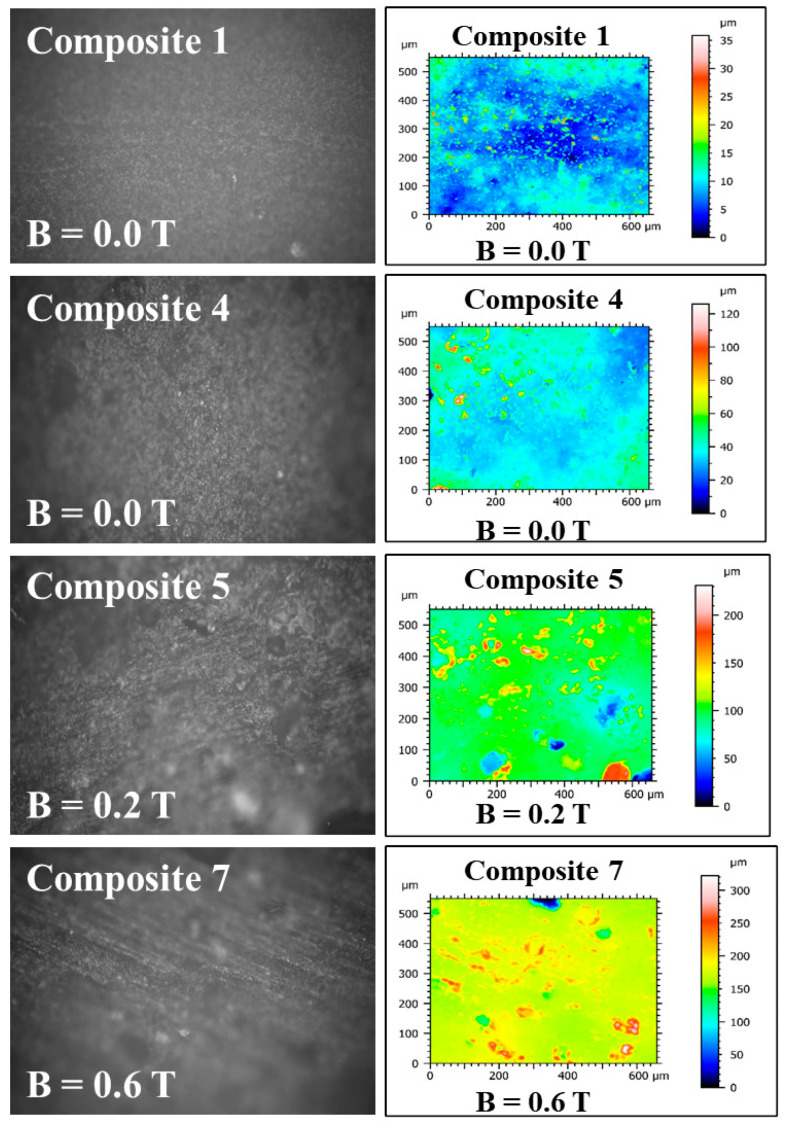
Images of the cross-sectional surfaces of composites No. 1, 4, 5 and 7 taken with the UP-3000 3D optical profilometer from Rtec-Instruments Inc. (San Jose, CA, USA). Taken at 20× magnification with a field of view of 860 μm × 650 μm, 0.31 m optical resolution μ, and 8 nm vertical resolution.

The third part concerned the change in parameters in polymer composites No. 1, 4, 5 and 7 in the top layer of the composite (Gumosil B), where admixtures are used, in this case with the use of *C. longa* and iron (Carbonyl iron), as a result of the additional action of a constant magnetic field (Figure 10).

Sample 1 consisted of “Gumosil B” silicone rubber alone, while samples 4, 5, and 7 were a mixture of “Gumosil B” silicone rubber, turmeric powder, and carbonyl iron. Samples 1 and 4 were not prepared in a magnetic field (B = 0 T), while samples 5 and 7 were in a magnetic field with an induction of B = 0.2 T and B = 0.6 T, respectively. Table 2 presents the values of roughness parameters for the individual samples.

**Figure 11:**
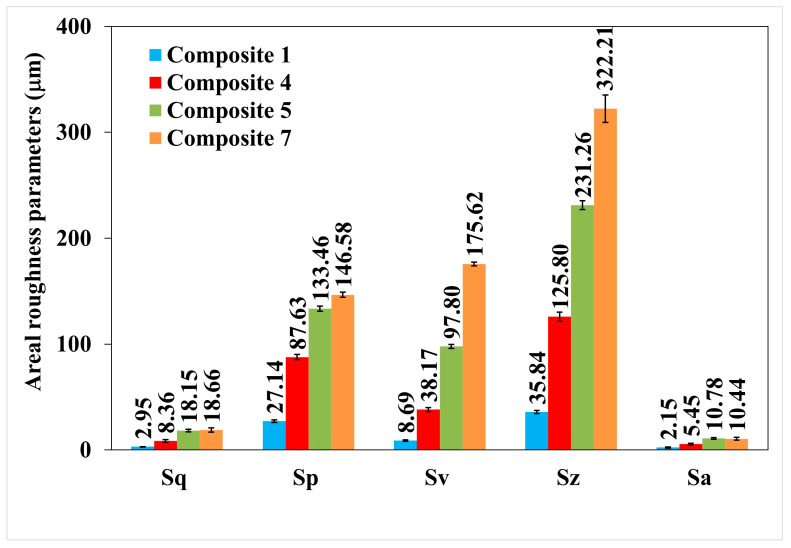
Surface roughness parameters of cross-sections of polymer composites numbered 1, 4, 5, and 7.

The changes in the surface roughness parameters of polymer composites No. 1, 4, 5, and 7 are also illustrated in Figure 11, prepared with and without a permanent magnetic field.

Figure 11 shows the values of the roughness parameters of samples No. 4 and 7, i.e., those obtained without a magnetic field (B = 0 T) and in a magnetic field (B = 0.6 T). All roughness parameters have increased in a constant magnetic field, which indicates an increase in the roughness of polymer composites.

In order to determine a statistical effect of magnetic field induction B on the surface roughness parameters of polymer composites 4, 5, and 7, an ANOVA analysis was performed. It was found that the *p*-value was lower than 0.05, which means that there are statistically significant differences between results. Thus, one can conclude that magnetic field induction B has a statistically significant effect on the roughness parameters of these composites.

## 3. Materials and Methods

### 3.1. Components of Polymeric Composites

**Figure 12:**
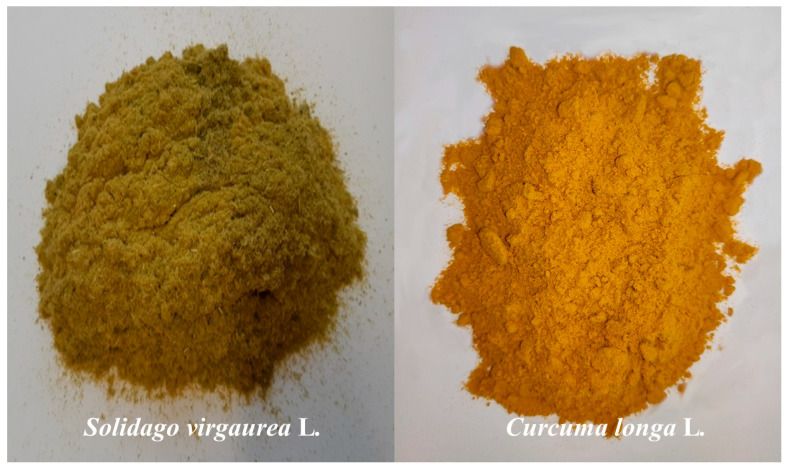
Plant admixtures used in two-layer polymer composites: *S. virgaurea* and *C. longa*.

The following components were used to prepare the two-layer polymer composites: silicone rubber “Polastosil M-56” (manufactured by Chemical Plant “Silikony Polskie” Sp. z o.o., Nowa Sarzyna, Poland), less flexible, constituting the basis of a two-layer composite; silicone rubber “Gumosil B” (manufactured by Chemical Plant “Silikony Polskie” Sp. z o.o., Nowa Sarzyna, Poland), more flexible, constituting the upper part of the composite, in which the admixtures used were placed; OL—1 hardener (manufactured by Chemical Plant “Silikony Polskie” Sp. z o.o., Nowa Sarzyna, Poland); plant admixtures such as *S. virgaurea*) (Figure 12), which is used in the treatment of urolithiasis, urinary tract inflammations, and gastroenteritis, containing phenolic glucosides, phenolic acids, and flavonoids with disinfectant and antibacterial properties; and *C. longa*, with anti-cancer, bactericidal, and antiviral properties, eliminating inflammation of the bile ducts and neutralizing free radicals.

### 3.2. Preparation of Test Samples

**Figure 13:**
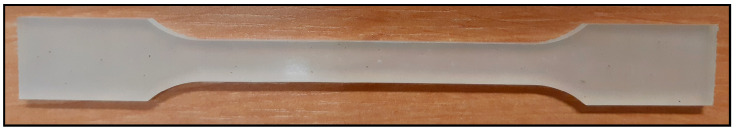
Molds for testing the mechanical tensile strength of polymer composites in accordance with DIN EN ISO 527-1.

The bottom layer of the composite was made of silicone rubber of “Polastosil M-56” type, with lower flexibility, containing no additives. This layer was the base of the two-layer composite. After developing the composition of the top layer of the composite containing the admixtures, the individual components were weighed in the following order: “Gumosil B”-type silicone rubber (characterized by greater flexibility), plant admixtures such as *S. virgaurea*) or *C. longa*, in the amount of 10% *w*/*w*; magnetic particles were added to some of them in the form of carbonyl iron in the amount of 20% *w*/*w* and OL—1 silicone rubber hardener in the amount of 3% in relation to the polymer amount. The composition of the tested samples is presented in Table 1. The tests of mechanical tensile strength of the polymer composites were carried out in accordance with the test standard: DIN EN ISO 527-1. The molds for these tests are shown in Figure 13.

### 3.3. Testing Methodology

The tensile strength was tested in accordance with the DIN EN ISO 527-1 and DIN EN ISO 527-2 standards. On the basis of these studies, a correction relationship was developed to correct the stress sought σ, which arises from the stretching of both polymer layers of the two-layer composite simultaneously, without the need to separate them. Thus, Equation (4), for the stress generated in a material, can be written as (Figure 2):

(4) $$\sigma =\frac{F}{P}=\frac{F}{b(h1+h2)}$$

If the entire layer of the composite was “Polastosil M-56”, i.e., h = h1, the stress σ reached the maximum and was equal to 1.706472 MPa. On the other hand, if the entire layer of the composite was “Gumosil B”, i.e., h = h2, the stress σ was minimal and equal to 0.275885 MPa. Thus, the equations for the maximum and minimum stresses generated in both silicone rubbers can be written as:

(5) $$\sigma max=\frac{Fmax}{b\cdot h1}$$

and

(6) $$\sigma min=\frac{Fmin}{b\cdot h2}$$

In Equations (4)–(6) there is one common parameter b, which is the width of the narrowed part of the composite sample. Comparing Equations (4)–(6) to one another, we obtain an equation, remembering that when using the equation for further calculations and applying units, we divide by “cm”:

(7) $$\frac{F}{\sigma (h1+h2)}=\frac{Fmax}{\sigma max\cdot h1}=\frac{Fmin}{\sigma min\cdot h2}$$

The stress σ formed in the material (composite) during the tension in a two-layer composite could be described by the equation:

(8) $$\sigma =\frac{F\cdot h2}{h1(h1+h2)}\cdot \frac{\sigma min\cdot Fmax}{\sigma max\cdot Fmin}$$

where

F—tensile force of a two-layer polymer composite;
Fmax—tensile force of single-layer silicone rubber “Polastosil M-56”;
Fmin—tensile force of single-layer silicone rubber “Gumosil B”;
h1—height of the bottom layer made of silicone rubber “Polastosil M-56”;
h2—height of the top layer made of silicone rubber “Gumosil B”, with or without additives;
σmin—tension forming during stretching in silicone rubber “Gumosil B”;
σmax—tension forming during stretching in silicone rubber “Polastosil M-56”.

The last part of the equation was a constant that could be calculated earlier, because the individual parameters describe the stresses and forces of both silicone rubbers without admixtures and they do not change. The constant for the studied example would therefore be as follows:

(9) $$const.=\frac{\sigma min\cdot Fmax}{\sigma max\cdot Fmin}$$

Using the results of the previously discussed and calculated tests, we were able to obtain the parameters we needed:

σmin = 0.275885 MPa = 2.813244 kG/cm^2^; σmax = 1.706472 MPa = 17.401171 kG/cm^2^; P1 = b · h = 10 · 4.49 = 44.9 mm^2^ = 0.449 cm^2^; P2 = b · h = 10 · 4.92 = 49.2 mm^2^ = 0.492 cm^2^; Fmin = σmin · P1 = 1.2631465 kG; Fmax = σmax · P2 = 8.5613761 kG.

Substituting the data into the last equation, we obtained the constant 1.0957683, which can be further used to calculate stresses in various two-layer polymer composites with the tested materials.

The surface contact angle was determined using the DSA 25E goniometer from KRUSS GmbH, Hamburg, Germany. The measurements consisted of measuring six measuring drops of liquids: water, diiodomethane, glycerin. On the basis of these measurements, the value of the surface contact angle of polymer composites was determined. Measurements of the surface roughness of cross-sections of the polymer composites were performed using the UP-3000 optical 3D profilometer from Rtec-Instruments Inc. (San Jose, CA, USA). Images were taken in the bright field mode, and surface roughness measurements were performed using the confocal microscopy mode. The images and surface roughness measurements were taken at 20× magnification with a field of view of 860 μm × 650 μm, an optical resolution of 0.31 μm, and vertical resolution of 8 nm. The surface roughness parameters defined in the ISO 25178 [ISO25178] standard were calculated using Digital Surf MountainsMap Imaging Topography software (Besançon, France). The water absorption *n_w_* of the sample was calculated from Equation (10):

(10) $$n_{w}=\frac{m_{n}-m}{m}\;\cdot 100\%$$

where *m_n_* is the mass of the sample saturated with distilled water (in g) and *m* is the mass of the dry sample (in g).

The frost resistance of the samples was tested as follows. The percentage of damage of the sample *S* was defined as the relative loss of its mass calculated from Equation (11):

(11) $$S=\frac{m_{1}-m_{2}}{m_{1}}\cdot 100\%$$

where *m*_1_ is the mass of the dried sample prepared for the test [g] and *m*_2_ is the mass of the sample dried after the end of the test [g].

The measurement of each sample for each property was repeated five times. The results presented in the graphs are the average of these five measurements, and their standard deviations were calculated using the STDEV.S built-in statistical function from Excel 365 (Microsoft Corporation, Redmond, WA, USA). The statistical analysis was carried out to demonstrate a statistically significant impact of magnetic field induction B, as an independent variable, on tensile stress, as well as its deformation during tensile strength, water absorbability, frost resistance, and contact angle as a dependent variable, of the composites under investigation. For this purpose, the one-way ANOVA test was performed using the ANOVA Single Factor function from the Analysis ToolPak add-in of Excel 365. The calculations were made at a 95% level of confidence (α = 0.05) on a dataset consisting of six groups in the case of the tensile stress, deformation during tensile strength, water absorbability, and frost resistance (magnetic induction B = 0.0, 0.2, 0.4, 0.6, 0.8, 1.0 T) parameters, or three groups in the case of the surface contact angle and surface roughness parameters (magnetic induction B = 0.0, 0.2, 0.6 T). Each group consisted of five elements (five measurement results at a given magnetic induction). The calculated significance probability (*p*-value) was used as a criterion for the occurrence of statistical differences between each group in a given dataset; namely, if *p*-value < 0.05, an independent variable impacts a dependent variable, otherwise, such a relationship does not occur.

A constant magnetic field with a magnetic induction B of (0.0–1.0 T) was generated using a laboratory electromagnet model LS-EM-7V with an LS-648 control power supply (all from Lake Shore Cryotronics, OH, USA).

## 4. Conclusions

The paper presents the results of research on new polymer composites based on two-layer polymer composites with favorable mechanical and functional properties. The bottom layer of the composite was a polymer with less elastic properties, i.e., a silicone rubber called “Polastosil M-56”, containing no admixtures. The top layer of the composite was a polymer with more elastic properties, i.e., a silicone rubber called “Gumosil B”, which contained plant admixtures in the amount of 10% *w*/*w* of goldenrod (*S. virgaurea*) or turmeric powder (*C. longa*) by mass. Some polymer composites additionally contained magnetic particles in the form of carbonyl iron (Fe) in the upper layer in the amount of 20% *w*/*w*. The mechanical tensile strength of the composites, water absorption, frost resistance and surface contact angle were tested. Microscopic examinations determined the roughness of the cross-sectional surfaces. A constant magnetic field with magnetic induction B, which was an additional external factor changing the properties and structure of two-layer polymer composites, was also used in the work. On the basis of the research, an additional relationship was developed to correct the stress sought σ, which arises during tension in a two-layer composite in both polymer layers of the composite simultaneously, without the need to separate them. The mechanical strength tests demonstrate that an increase in the induction of the magnetic field B from 0 T to 0.6 T during the polymerization process of the composites caused an increase in tensile stress (σ_m_) in the polymer composite and at the same time caused a decrease in the deformation (ε_m_) of the composite. Increasing the value of magnetic induction B during polymerization resulted in an increase in the water absorption (n_w_) of polymer composites with the addition of turmeric powder (10% *w*/*w*). and carbonyl iron (20%). The arrangement of the composite particles in the direction of the magnetic induction vector resulted in the appearance of additional spaces for water to penetrate into the composite. However, at the same time, the increase in the value of magnetic induction B resulted in an improvement in the frost resistance of polymer composites, i.e., a reduction in their weight loss during cyclic freezing and thawing. A constant magnetic field of B = 0.2 or 0.6 T also reduced the water contact angle of the polymer composites, which, in accordance with the principle, made them more hydrophilic. The above is consistent with the water absorption studies of polymer composites. All the roughness parameters of polymer composites, Sq—root mean square height of the surface, Sp—maximum height of peaks, Sv—maximum height of valleys, Sz—maximum height of the surface, and Sa—arithmetical mean height of the surface, were increased when tested in a constant magnetic field, both in the lower and upper layers, which indicated an increase in the roughness of polymer composites. The ANOVA test revealed the statistically significant impact of magnetic field induction B on properties of the tested composites, such as tensile stress, deformation during tensile strength, water absorbability, frost resistance, contact angle, and surface roughness parameters.

## Figures and Tables

**Table 1:** A list of the tested polymer composites on the basis of silicone rubber “Gumosil B”.

Composite Number	Magnetic Induction (T)	The Content of the Composite
1	0	silicone rubber “Gumosil B”
2	0	silicone rubber “Gumosil B”, *S. virgaurea*,
3	0	silicone rubber “Gumosil B”, *C. longa*,
4	0	silicone rubber “Gumosil B”, *C. longa*, carbonyl iron,
5	0.2	silicone rubber “Gumosil B”, *C. longa*, carbonyl iron,
6	0.4	silicone rubber “Gumosil B”, *C. longa*, carbonyl iron,
7	0.6	silicone rubber “Gumosil B”, *C. longa*, carbonyl iron,
8	0.8	silicone rubber “Gumosil B”, *C. longa*, carbonyl iron,
9	1.0	silicone rubber “Gumosil B”, *C. longa*, carbonyl iron,

**Table 2:** Roughness parameters for samples No. 1, 4, 5, and 7 (mean values with standard deviation).

Composite Number	Magnetic Induction (T)	Surface Roughness Parameters (µm)				
		Sq ± SD	Sp ± SD	Sv ± SD	Sz ± SD	Sa ± SD
1	0	2.95 ± 0.10	27.14 ± 1.13	8.69 ± 0.66	35.84 ± 1.50	2.15 ± 0.64
4	0	8.36 ± 1.23	87.63 ± 4.52	38.17 ± 1.91	125.80 ± 4.26	5.45 ± 1.00
5	0.2	18.15 ± 1.25	133.46 ± 2.42	97.80 ± 1.90	231.26 ± 4.25	10.78 ± 0.89
7	0.6	18.66 ± 2.01	146.58 ± 2.37	175.62 ± 1.69	322.21 ± 12.91	10.44 ± 1.31

## Data Availability

The original contributions presented in this study are included in the article. Further inquiries can be directed to the corresponding author.
